# Intestinal Capillariasis in a South Indian Farmer: A Report of a Rare Case From a Tertiary Care Hospital

**DOI:** 10.7759/cureus.110590

**Published:** 2026-06-10

**Authors:** Pavani Chinnapaka, Sri Lakshmi Kothakapa, Sulekha Ramireddy, Sanjay Reddy Thanugundla, Muralidhar Chinnapaka

**Affiliations:** 1 General Medicine, Employees' State Insurance (ESI) Hospital Nacharam, Hyderabad, IND; 2 Internal Medicine, Malla Reddy Institute of Medical Sciences, Hyderabad, IND; 3 Pharmacology, Government Medical College and Hospital Maheshwaram, Hyderabad, IND

**Keywords:** capillaria philippinensis, chronic diarrhoea, farmer, intestinal capillariasis, mebendazole, parasitic infection, south india, stool microscopy

## Abstract

Intestinal capillariasis is an uncommon food-borne helminthic infection caused by *Capillaria philippinensis*. It is classically reported from Southeast Asian countries, but sporadic cases from non-endemic regions, including India, show that the disease may be overlooked in routine clinical practice. We report the case of a 35-year-old male farmer from South India who presented with six months of chronic watery diarrhoea, abdominal pain, and marked weight loss. Clinical examination suggested malnutrition, and laboratory evaluation showed mild anaemia and eosinophilia. Stool microscopy revealed characteristic oval, thick-shelled eggs consistent with *Capillaria philippinensis*, confirming the diagnosis of intestinal capillariasis. The patient received oral mebendazole at a dose of 200 mg twice daily for 20 days, together with adequate hydration, dietary advice, and nutritional supplementation. During follow-up, there was a clear reduction in stool frequency, improvement in appetite, and gradual recovery of general health. Repeat stool microscopy performed after one month did not reveal any parasitic eggs, indicating parasitological clearance. This case emphasises that intestinal capillariasis should be considered as a possible diagnosis in patients with long-standing diarrhoea, weight loss, and eosinophilia, even in areas where the disease is not commonly reported. Early stool examination, careful dietary history, and timely anti-helminthic therapy can prevent prolonged morbidity and possible complications.

## Introduction

Intestinal capillariasis is a rare but clinically important parasitic disease caused by the nematode *Capillaria philippinensis *[1-8]. The parasite was first described in the Philippines [8] and has since been reported from Taiwan [3,4], Thailand [5-7], India [9], Indonesia [10], Korea [11], China [12], and other regions [1-9]. Although it remains uncommon in India, previous Indian reports indicate that the disease can occur in non-endemic settings and may be missed when stool microscopy is not carefully performed [9].

The infection is most commonly acquired after eating raw, undercooked, dried, pickled, or otherwise inadequately processed small freshwater or brackish-water fish that contain infective larvae [10]. Humans are considered accidental hosts, as the natural cycle of *Capillaria philippinensis* usually involves fish-eating birds and aquatic hosts [11]. After ingestion, the larvae reach the small intestine, mature into adult worms, and become embedded in the intestinal mucosa [12,13]. This mucosal involvement interferes with normal absorption and may lead to persistent watery diarrhoea, abdominal discomfort, malabsorption, protein-losing enteropathy, electrolyte imbalance, progressive weight loss, and severe wasting when diagnosis and treatment are delayed [14-16].

A clinically important feature of intestinal capillariasis is its ability to sustain infection through autoinfection. In this process, some eggs may develop within the intestine and release larvae before being passed out in stool. These larvae can mature further within the same host, allowing the parasite burden to increase without the need for repeated external exposure. This explains why some patients develop prolonged or progressively worsening symptoms over weeks to months. Autoinfection may also contribute to severe nutritional depletion, hypoalbuminaemia, fluid and electrolyte disturbances, and occasionally life-threatening illness if the condition is not recognised early. Therefore, in patients with chronic diarrhoea, weight loss, eosinophilia, and a history of possible fish exposure, early stool examination and timely anti-helminthic therapy are essential to interrupt the cycle of infection and prevent complications [2,6].

The clinical presentation can resemble many common gastrointestinal disorders, including chronic infective diarrhoea, inflammatory bowel disease (IBD), coeliac disease, intestinal tuberculosis (ITB), tropical sprue, and malignancy-related cachexia. In resource-limited or non-endemic settings, the diagnosis is often delayed because clinicians may not initially suspect this parasite. Demonstration of eggs, larvae, or adult worms in stool remains the most practical diagnostic approach [2,3,6]. We present a rare case of intestinal capillariasis in a South Indian farmer diagnosed by stool microscopy and successfully treated with mebendazole.

## Case presentation

A 36-year-old male Indian farmer from Narketpally, Nalgonda district, Telangana, attended the Department of General Medicine at a tertiary care hospital in South India with a history of recurrent watery diarrhoea for nearly six months. The diarrhoea was non-bloody and occurred repeatedly, causing considerable discomfort and a gradual decline in his general health. It was associated with intermittent cramp-like abdominal pain, poor appetite, easy fatigability, progressive weakness, and an unintentional weight loss of approximately 8 kg over six months. He did not report fever, blood or mucus in stool, jaundice, vomiting, or features suggestive of acute intestinal obstruction. There was no known history of diabetes mellitus, TB, IBD, chronic liver disease, renal disease, or any other long-standing systemic illness. He denied any travel outside his native region, which made an imported source of infection less likely during the initial assessment. On further enquiry into his dietary habits, he reported frequent consumption of locally available small freshwater fish, particularly from nearby rural markets and local water sources. He also mentioned that, on some occasions, the fish was eaten after minimal cooking or inadequate processing. Although the exact source of infection could not be confirmed, this dietary history suggested a possible local food-borne exposure and added epidemiological relevance to the diagnosis in a non-endemic setting.

On clinical examination, the patient appeared thin, tired, and chronically ill, with features suggestive of nutritional depletion rather than an acute infective episode. He had visible temporal wasting and reduced subcutaneous fat. His height was 168 cm and weight was 48 kg, with a calculated BMI of 17.0 kg/m², indicating undernutrition. At presentation, he was afebrile and haemodynamically stable, with a pulse rate of 84 beats/min, blood pressure of 116/74 mmHg, respiratory rate of 18 breaths/min, and oxygen saturation of 98% on room air. There was no obvious icterus or clinically significant lymphadenopathy. Abdominal examination did not reveal guarding, rigidity, organomegaly, or signs of an acute surgical abdomen. The overall clinical picture of prolonged watery diarrhoea, reduced appetite, weight loss, weakness, and nutritional decline raised the possibility of a chronic intestinal infection or malabsorption syndrome.

Baseline investigations showed mild anaemia, with haemoglobin of 10.8 g/dL and MCV of 78 fL. The total leukocyte count was within normal limits; however, the absolute eosinophil count was elevated at 950 cells/µL, supporting a possible parasitic aetiology. Biochemical evaluation showed mild hyponatraemia (serum sodium 132 mmol/L), mild hypokalaemia (serum potassium 3.3 mmol/L), and reduced serum albumin (3.0 g/dL). These findings were consistent with the effects of prolonged diarrhoeal illness, nutritional depletion, and possible intestinal protein loss.

In view of the long duration of symptoms and associated weight loss, stool microscopy was performed as an important first-line investigation. Stool examination was initially performed using a direct saline and iodine wet mount. Given the chronicity of symptoms and eosinophilia, the sample was further processed using the formalin-ether concentration method to improve parasitic egg detection. Microscopy revealed oval, thick-shelled eggs with bipolar plug-like ends and granular internal contents, morphologically suggestive of *Capillaria philippinensis*. The presence of these characteristic eggs, when interpreted along with the patient’s chronic watery diarrhoea, weight loss, eosinophilia, and nutritional decline, supported the diagnosis of intestinal capillariasis. This finding was clinically important because the disease can easily be missed if stool examination is not carefully performed or if rare helminthic infections are not considered.

The patient was managed on an outpatient basis and treated with oral mebendazole 200 mg twice daily for 20 days. Mebendazole is a broad-spectrum anti-helminthic drug that acts by inhibiting tubulin polymerisation and microtubule formation in intestinal nematodes. This disrupts glucose uptake and energy metabolism of the parasite, leading to progressive immobilisation and death of the worm. Supportive care in the form of hydration, dietary advice, and nutritional supplementation was also provided. Following treatment, the patient showed steady clinical improvement. His stool frequency reduced, abdominal discomfort subsided, appetite improved, and his general condition became better. At one-month follow-up, repeat stool microscopy did not show parasitic eggs, suggesting parasitological clearance. At one-month follow-up, repeat stool microscopy was negative for parasitic eggs. Repeat CBC also showed improvement, with haemoglobin increasing from 10.8 g/dL to 11.6 g/dL and absolute eosinophil count decreasing from 950 cells/µL to 420 cells/µL, supporting clinical and haematological recovery after treatment The clinical course of the patient is summarised in Table 1.

**Table 1:** Clinical profile, diagnostic findings, and treatment outcome of the present case

Parameter	Findings
Age and sex	36-year-old male
Occupation	Farmer
Geographical region	South India
Duration of illness	Six months
Presenting symptoms	Chronic watery diarrhoea, intermittent abdominal pain, reduced appetite, easy fatigability, progressive weakness, and unintentional weight loss of approximately 8 kg
Travel history	No history of travel outside the native region
Dietary history	Frequent consumption of locally available small freshwater fish, occasionally eaten in inadequately cooked form
General condition at presentation	Thin built, tired-looking, and clinically undernourished
Baseline haematological findings	Mild anaemia with haemoglobin 10.8 g/dL and MCV 78 fL; total leukocyte count within normal limits; absolute eosinophil count elevated at 950 cells/µL
Baseline biochemical findings	Mild hyponatraemia with serum sodium 132 mmol/L, mild hypokalaemia with serum potassium 3.3 mmol/L, and reduced serum albumin 3.0 g/dL
Viral serology	Non-reactive
Diagnostic method	Stool microscopy using direct saline and iodine wet mount, followed by formalin-ether concentration method
Parasitological finding	Oval, thick-shelled eggs with bipolar plug-like ends and granular internal contents, morphologically suggestive of Capillaria philippinensis
Final diagnosis	Intestinal capillariasis
Treatment given	Oral mebendazole 200 mg twice daily for 20 days, along with hydration, dietary advice, and nutritional supplementation
Follow-up outcome	Symptomatic improvement with reduction in stool frequency, better appetite, improved general condition, and repeat stool microscopy negative for parasitic eggs after one month

**Figure 1:**
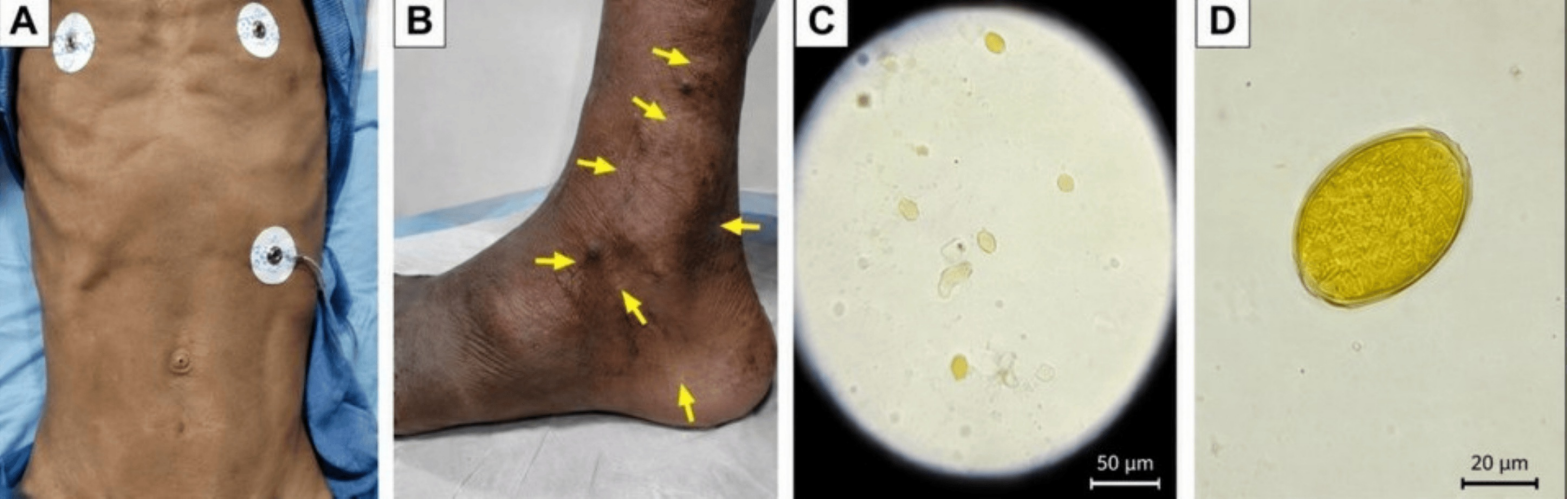
Clinical and microscopic findings in intestinal capillariasis (A) Clinical image showing a thin abdominal profile at the time of examination; (B) lower limb image showing visible skin changes and features of nutritional wasting, highlighted with yellow arrows; (C) stool microscopy demonstrating multiple oval, yellowish parasitic eggs in the microscopic field, with scale bar; (D) high-power microscopic image showing a single thick-walled oval egg with granular internal contents, morphologically suggestive of *Capillaria philippinensis*, with scale bar.

The clinical and microscopic findings are shown in Figure 1. The clinical images demonstrate the patient’s thin abdominal profile and lower limb changes, reflecting the chronic nutritional impact of the illness. Stool microscopy at low power showed multiple oval yellowish parasitic eggs in the field, while the high-power image demonstrated a single thick-walled oval egg with granular internal contents, consistent with the morphology of *C. philippinensis.*

In a patient presenting with prolonged diarrhoea and significant weight loss, a broad range of differential diagnoses must be considered. Common possibilities include intestinal tuberculosis, inflammatory bowel disease, tropical sprue, coeliac disease, chronic amoebiasis, strongyloidiasis, gastrointestinal malignancy, and other causes of malabsorption. In the present case, the diagnosis became clearer after stool microscopy demonstrated parasitic eggs compatible with intestinal capillariasis, helping to distinguish it from other chronic gastrointestinal disorders.

## Discussion

Intestinal capillariasis is a rare but clinically significant helminthic infection that may cause prolonged morbidity when not recognised early. The causative organism, *Capillaria philippinensis*, is a small nematode that predominantly affects the small intestine. Human infection is generally acquired through ingestion of raw, undercooked, or inadequately processed small freshwater or brackish-water fish harbouring infective larvae [1,2]. Although the disease has been reported more frequently from Southeast Asian countries, sporadic cases from other geographical regions show that it should not be regarded as a disease limited to classical endemic areas [3-5]. The present case is therefore noteworthy, as it describes intestinal capillariasis in a South Indian farmer who had no history of travel outside his native region. This supports the possibility that local exposure may be sufficient for infection in selected cases.

The clinical features in this patient were consistent with chronic intestinal parasitic disease. He presented with watery diarrhoea for six months, intermittent abdominal pain, reduced appetite, progressive weakness, unintentional weight loss, mild anaemia, and eosinophilia. Similar clinical patterns have been documented in previous reports, where patients commonly presented with persistent diarrhoea, abdominal discomfort, anorexia, weight loss, hypoalbuminaemia, and features of nutritional depletion [2,6-8]. In intestinal capillariasis, symptoms may become prolonged because the parasite has the ability to maintain infection within the host through internal autoinfection. In this process, some eggs may embryonate and release larvae within the intestine itself, allowing further maturation of the parasite without repeated external exposure. This can gradually increase the worm burden and worsen intestinal damage, resulting in malabsorption, protein loss, electrolyte imbalance, and progressive wasting if treatment is delayed [2,6].

The diagnosis in this case was made by stool microscopy, which remains the most practical and widely available diagnostic method in routine clinical settings. Identification of oval, thick-shelled eggs with morphology suggestive of *C. philippinensis* provided direct evidence of parasitic infection. The eggs are generally described as oval or peanut-shaped, with a striated shell and bipolar plug-like ends, although their appearance may vary depending on the stage of development and the method of stool preparation [1,2,9]. In the present case, stool examination was performed using direct wet mount and formalin-ether concentration, which improved the likelihood of detecting parasitic eggs. Since egg shedding may be scanty or intermittent, a single negative stool examination does not reliably exclude the infection. Therefore, repeated stool examinations should be considered when clinical suspicion remains high [2,6].

This case also illustrates why intestinal capillariasis can be missed in day-to-day clinical practice. Chronic diarrhoea with weight loss has a broad differential diagnosis, especially in tropical regions. Intestinal tuberculosis, inflammatory bowel disease, tropical sprue, coeliac disease, chronic amoebiasis, strongyloidiasis, and gastrointestinal malignancy may all present with overlapping symptoms. In such situations, stool microscopy is a simple but highly useful investigation, as it can provide a direct parasitological diagnosis when ova, larvae, or adult forms are detected. The presence of eosinophilia in this patient added further support for a parasitic cause, although eosinophilia is not diagnostic by itself and must be interpreted along with dietary history, clinical findings, and microbiological evidence.

The patient was treated with oral mebendazole 200 mg twice daily for 20 days, along with hydration, dietary advice, and nutritional supplementation. Mebendazole is a broad-spectrum anti-helminthic agent that acts by interfering with microtubule formation in intestinal nematodes, thereby impairing glucose uptake and energy metabolism in the parasite. This ultimately leads to immobilisation and death of the worm. Following treatment, the patient showed symptomatic improvement, and repeat stool microscopy after one month was negative for parasitic eggs. Both mebendazole and albendazole have been reported to be effective in intestinal capillariasis [2,6]. Early initiation of therapy is important because untreated disease can progress to severe malnutrition, hypoalbuminaemia, electrolyte disturbances, protein-losing enteropathy, and, rarely, fatal outcomes [2,7,8].

The patient's rural background and occupation as a farmer may have epidemiological relevance. People living in rural settings may have greater contact with local water sources, fish handling, and traditional food practices. In the present case, he reported frequent consumption of locally available small freshwater fish, occasionally in inadequately cooked form. Although a definite source of infection could not be confirmed, this dietary history provides a plausible route of exposure. Such uncertainty is common in sporadic cases because the infective exposure may have occurred weeks or months before diagnosis, and patients may not recall exact details of food preparation. Therefore, careful questioning about consumption of raw, undercooked, dried, pickled, or fermented fish is important in patients with chronic diarrhoea and suspected parasitic disease [2,5,6].

From a public health perspective, this case highlights the importance of food safety, safe water practices, and awareness regarding fish-borne parasitic infections. Avoidance of raw or inadequately cooked fish, proper cooking methods, improved sanitation, and health education in rural communities may help reduce the risk of transmission. In regions where chronic diarrhoeal illnesses are common, clinicians and laboratory personnel should remain alert to uncommon parasitic causes. Use of stool concentration methods, along with careful microscopic examination, can improve the detection of unusual ova that may otherwise be overlooked.

Overall, this case reinforces the need to consider intestinal capillariasis in patients presenting with long-standing watery diarrhoea, weight loss, eosinophilia, and nutritional decline, even in non-endemic regions such as India, where the infection is not commonly reported. The diagnosis is usually straightforward when the possibility is considered, and stool microscopy is performed carefully. Timely anti-helminthic therapy, combined with supportive and nutritional care, can result in complete clinical and parasitological recovery and prevent avoidable complications.

## Conclusions

Intestinal capillariasis is an uncommon but treatable cause of chronic diarrhoea, abdominal pain, weight loss, and nutritional decline. This case in a South Indian farmer highlights that the infection may occur even outside traditionally recognised endemic areas. Careful stool microscopy remains a simple and useful diagnostic method, particularly when chronic diarrhoea is associated with eosinophilia and nutritional depletion. Early diagnosis followed by appropriate treatment with mebendazole can lead to both clinical improvement and parasitological clearance. In patients with prolonged watery diarrhoea, weight loss, eosinophilia, and a history of consuming raw, undercooked, or inadequately processed fish, intestinal capillariasis should be included in the differential diagnosis, even in non-endemic settings.
